# Differential Tumor Response and Conversion Outcomes Associated with First-Line Biologic Strategies in Liver-Limited *RAS* Wild-Type Metastatic Colorectal Cancer

**DOI:** 10.32604/or.2026.085229

**Published:** 2026-08-13

**Authors:** Shih-Wei Chiang, Ming-Cheng Chen, Chang-Lin Lin, Yi-Lin Huang, Feng-Fan Chiang, Shun-Fa Yang

**Affiliations:** 1 Institute of Medicine, Chung Shan Medical University, Taichung, Taiwan; 2 Division of Colorectal Cancer, Department of Surgery, Taichung Veterans General Hospital, Taichung, Taiwan; 3 School of Medicine, National Yang Ming Chiao Tung University, Taipei, Taiwan; 4 College of Humanities and Social Sciences, Providence University, Taichung, Taiwan; 5 Department of Medical Research, Chung Shan Medical University Hospital, Taichung, Taiwan

**Keywords:** Metastatic colorectal cancer, *RAS* wild-type, panitumumab, cetuximab, bevacizumab, conversion surgery

## Abstract

**Background:** Anti-EGFR therapy is widely used as first-line treatment for *RAS* wild-type metastatic colorectal cancer (mCRC), particularly in patients with left-sided tumors. In liver-limited disease, maximizing tumor shrinkage may facilitate conversion to resectability; however, comparative real-world evidence among panitumumab, cetuximab, and bevacizumab remains limited. This study aimed to compare the clinical outcomes of these biologic agents in patients with *RAS* wild-type mCRC. **Methods:** We retrospectively analyzed 241 patients with *RAS* wild-type mCRC treated with first-line chemotherapy plus panitumumab (*n* = 76), cetuximab (*n* = 80), or bevacizumab (*n* = 85) between 2016 and 2024. Outcomes included depth of response (DpR), objective response rate (ORR), disease control rate (DCR), conversion surgery rate, progression-free survival (PFS), and overall survival (OS). **Results:** Overall, panitumumab demonstrated superior tumor response, achieving the highest ORR (69.7%) and conversion surgery rate (34.2%), although PFS and OS were comparable among treatment groups. In patients with liver-limited disease, panitumumab was associated with a higher conversion surgery rate than bevacizumab (50.0% vs. 19.4%) and a trend toward longer OS. Right-sided primary tumors and *BRAF* mutation remained independent predictors of poorer OS. **Conclusions:** Panitumumab-based therapy was associated with deeper tumor shrinkage and higher conversion rates, particularly in liver-limited mCRC. These findings suggest that greater cytoreduction may increase opportunities for local treatment in selected patients. However, given the retrospective nature of the study, the findings should be considered hypothesis-generating and require prospective validation.

## 1 Introduction

Metastatic colorectal cancer (mCRC) is a biologically heterogeneous disease in which therapeutic decision-making is increasingly informed by molecular alterations, primary tumor sidedness, and the extent and distribution of metastatic involvement [1,2,3]. In patients with *RAS* wild-type tumors, anti-epidermal growth factor receptor (EGFR)-based therapy has become an established first-line option, particularly for left-sided disease, where it has demonstrated high response rates and favorable survival outcomes in randomized trials and pooled analyses [2,4,5,6,7].

For patients with liver-limited metastatic disease, however, the therapeutic objective extends beyond disease control alone. In selected cases, effective induction therapy may substantially reduce tumor burden and enable conversion to potentially curative-intent liver resection or other metastasis-directed local therapies [8,9,10]. In this setting, the quality of radiologic response is of particular clinical relevance, because early tumor shrinkage and depth of response have been associated with resectability and long-term outcome [11]. Therefore, treatment efficacy in liver-limited mCRC should not be evaluated solely on the basis of progression-free survival, but also in terms of cytoreductive potential and the opportunity to achieve secondary local treatment.

Although the efficacy of anti-EGFR therapy in *RAS* wild-type metastatic colorectal cancer has been established in prospective randomized trials, direct prospective first-line head-to-head data comparing cetuximab and panitumumab remain limited, particularly in clinically selected populations such as patients with liver-limited disease. In contrast, accumulating real-world evidence has begun to address this gap. A nationwide Taiwanese propensity overlap weighting analysis comparing bevacizumab, cetuximab, and panitumumab suggested more favorable outcomes with anti-EGFR-based therapy, especially in left-sided tumors, and further highlighted the potential role of panitumumab in facilitating conversion treatment [12]. Similarly, a nationwide database study directly comparing cetuximab and panitumumab found broadly comparable overall survival and conversion surgery outcomes between the two agents, while underscoring the prognostic importance of metastasectomy in routine practice [13]. More recently, a multicenter real-world observational study from the Japanese Society for Cancer of the Colon and Rectum provided additional first-line comparative data for cetuximab- and panitumumab-based doublet chemotherapy in left-sided colorectal cancer [14]. Other observational studies have likewise shown that treatment effectiveness in daily practice is strongly influenced by primary tumor sidedness, metastatic extent, resectability assessment, and institutional multidisciplinary decision-making, factors that are often less standardized than in clinical trials [15,16]. Therefore, real-world analyses from individual institutions remain clinically meaningful, as they may better capture local patterns of patient selection, imaging intervals, and conversion-to-local-therapy strategies, thereby complementing evidence from randomized studies and large administrative databases.

Among available anti-EGFR agents, both panitumumab and cetuximab are widely used in clinical practice [17]. Nevertheless, comparative data between these agents in real-world first-line treatment remain limited, and comparisons between anti-EGFR-based and bevacizumab-based induction strategies remain clinically important when conversion to local therapy is a realistic goal [5,6,7,9,10,18].

In the present study, we evaluated a retrospective real-world cohort of patients with *RAS* wild-type mCRC who received first-line chemotherapy plus panitumumab, cetuximab, or bevacizumab. We compared treatment response and survival outcomes across these groups, with a particular focus on patients with liver-limited metastases. We hypothesized that panitumumab-based therapy would be associated with deeper tumor shrinkage, higher conversion-to-surgery rates, and more favorable long-term survival in this clinically important subgroup.

## 2 Materials and Methods

### 2.1 Study Design and Patient Selection

**Figure 1:**
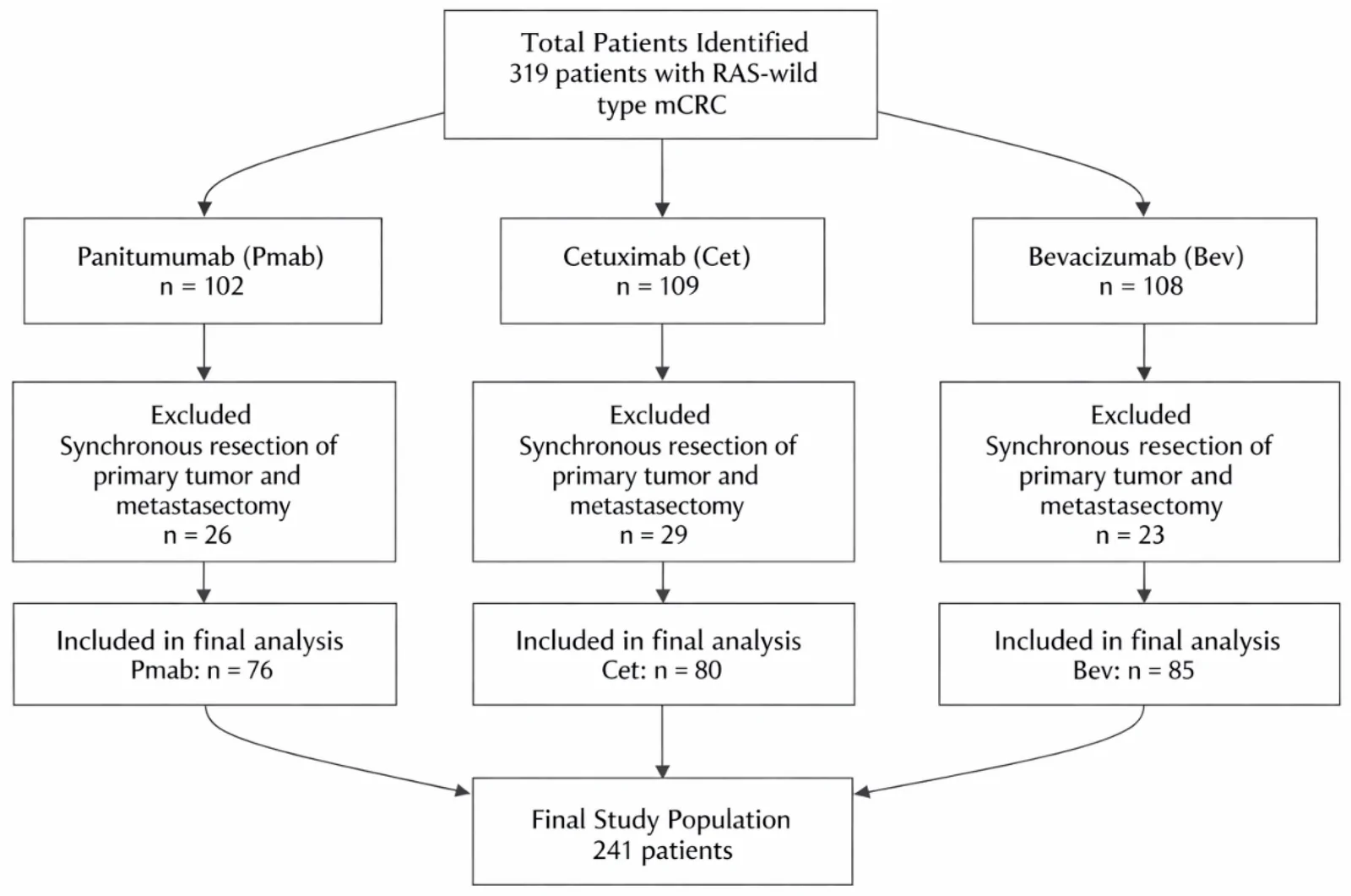
**Study flowchart**. Selection of patients with RAS wild-type metastatic colorectal cancer included in the study. Of 319 initially identified patients, 78 who underwent synchronous resection of the primary tumor and metastasectomy were excluded, leaving 241 patients for the final analysis: panitumumab, *n* = 76; cetuximab, *n* = 80; bevacizumab, *n* = 85.

This was a retrospective cohort study conducted at Taichung Veterans General Hospital, a tertiary referral medical center in Taiwan. Patients with *RAS* wild-type metastatic colorectal cancer who received first-line chemotherapy in combination with panitumumab, cetuximab, or bevacizumab from 1 January 2016, through 31 December 2024 were identified from the institutional database. Patients undergoing synchronous resection of the primary tumor and metastatic lesions were excluded to focus specifically on patients requiring induction systemic therapy for initially unresectable or potentially convertible metastatic disease. Among 319 initially screened patients, 102 received panitumumab-based therapy, 109 received cetuximab-based therapy, and 108 received bevacizumab-based therapy (Fig. 1). Following exclusion of 78 patients who underwent synchronous primary tumor resection and metastasectomy, 241 patients remained eligible for analysis, including 76 in the panitumumab group, 80 in the cetuximab group, and 85 in the bevacizumab group.

### 2.2 Data Collection

Baseline clinicopathologic variables were collected from the institutional database and medical records, including age, sex, Eastern Cooperative Oncology Group (ECOG) performance status, primary tumor sidedness, mismatch repair (MMR) status, *BRAF* mutation status, sites of metastasis, prior primary tumor resection, year of treatment initiation, and chemotherapy backbone. For patients with liver metastases, additional liver-specific variables were recorded, including the number of liver metastases, largest liver metastasis diameter (cm), presence of metachronous versus synchronous liver metastasis, and bilobar liver involvement. This retrospective study was approved by the Institutional Review Board of Taichung Veterans General Hospital (IRB No. SE23223C). The requirement for informed consent was waived by the Institutional Review Board because of the retrospective nature of the study. All procedures were performed in accordance with the ethical standards of the institutional research committee and the principles of the Declaration of Helsinki.

### 2.3 Definitions

For subgroup analyses, liver-limited metastasis was defined as metastatic disease confined to the liver at the initiation of first-line systemic therapy. Conversion surgery was defined as metastasis-directed surgery performed after initial systemic treatment with the intention of achieving complete macroscopic resection of metastatic disease. Patients underwent regular follow-up with contrast-enhanced computed tomography (CT), magnetic resonance imaging (MRI), or positron emission tomography/computed tomography (PET/CT), as clinically indicated. Survival status and clinical outcomes were obtained from the electronic medical records of Taichung Veterans General Hospital, including outpatient records, hospitalization records, imaging reports, and follow-up documentation. Overall survival (OS) was defined as the interval from initiation of first-line treatment to death from any cause or last follow-up. Progression-free survival (PFS) was defined as the interval from initiation of first-line treatment to documented disease progression or death from any cause, whichever occurred first.

Tumor response was assessed according to the Response Evaluation Criteria in Solid Tumors (RECIST), version 1.1 [19]. Objective response rate (ORR) was defined as the proportion of patients who achieved a complete response (CR) or partial response (PR) as their best overall response. Disease control rate (DCR) was defined as the proportion of patients who achieved CR, PR, or stable disease (SD). Depth of response (DpR) was defined as the maximal percentage reduction in tumor burden from baseline to nadir during first-line treatment, based on the sum of the longest diameters of target lesions. DpR was calculated as: DpR (%) = [(baseline sum of target lesion diameters − nadir sum of target lesion diameters)/baseline sum of target lesion diameters] × 100.

*RAS* mutational status was determined using allele-specific real-time polymerase chain reaction (PCR)-based assays performed in the Department of Pathology at Taichung Veterans General Hospital. Extended *RAS* testing included *KRAS* and *NRAS* exons 2, 3, and 4 (codons 12, 13, 59, 61, 117, and 146). Analyses were performed on formalin-fixed paraffin-embedded tumor tissue using the cobas z 480 analyzer (Roche Diagnostics, Basel, Switzerland). Patients were classified as *RAS* wild-type only when no detectable pathogenic mutations were identified within the tested *KRAS* and *NRAS* regions. MMR testing was not routinely performed for all patients during the study period and was generally requested at the discretion of the treating physician when clinically indicated. Therefore, MMR status was unavailable in a subset of patients and was recorded as “unknown”.

### 2.4 Treatment and Response Assessment

All patients received first-line chemotherapy in combination with panitumumab, cetuximab, or bevacizumab according to physician decision and multidisciplinary discussion in routine clinical practice. Radiologic response was evaluated using serial cross-sectional imaging, including contrast-enhanced computed tomography or magnetic resonance imaging, as available in routine care. To improve the consistency of response assessment in this retrospective cohort, imaging studies were independently reviewed by two colorectal surgeons, Shih-Wei Chiang and Ming-Cheng Chen, according to RECIST version 1.1. Any discrepancies were resolved through joint review and consensus. Interobserver agreement was not formally assessed using Cohen’s kappa or other statistical measures. Because imaging intervals were not fully standardized in this real-world cohort, early tumor shrinkage was not analyzed. Instead, treatment activity was evaluated using best overall radiologic response, ORR, DCR, DpR, and conversion surgery rate.

### 2.5 Conversion Surgery Assessment

Resectability assessment and conversion treatment decisions were performed through a multidisciplinary team (MDT) approach involving colorectal surgeons, hepatobiliary (HPB) surgeons, medical oncologists, radiologists, pathologists, and other oncology care team members. Patients generally underwent imaging reassessment every 3 months according to institutional practice and guideline-based follow-up protocols. Cases considered potentially resectable after systemic therapy were formally discussed during MDT meetings, and final surgical resectability was determined by the HPB surgeon based on technical feasibility and multidisciplinary consensus. MDT discussions and treatment decisions were routinely documented within the institutional medical record system.

### 2.6 Statistical Analysis

Baseline characteristics were summarized by treatment group (panitumumab, cetuximab, bevacizumab). Continuous variables were reported as medians with interquartile ranges (IQR) and compared using the Kruskal–Wallis test. Categorical variables were expressed as counts (percentages) and compared using the chi-square or Fisher’s exact test, as appropriate. Overall survival (OS) and progression-free survival (PFS) were estimated with the Kaplan–Meier method and compared using the log-rank test. Prognostic factors were assessed with Cox proportional hazards models; clinically relevant variables were entered into multivariable models (enter method), with results reported as hazard ratios (HRs) and 95% confidence intervals (CIs). Variables with *p* < 0.10 in univariable analyses together with clinically relevant covariates identified a priori were entered into the multivariable Cox regression models. Covariates were selected according to a prespecified conceptual framework in which patient fitness, tumor biology, disease burden, treatment era, and chemotherapy backbone were considered potential confounders of treatment selection and survival outcomes. Objective response rate (ORR), disease control rate (DCR), and conversion surgery rate were compared using logistic regression, reported as odds ratios (ORs) with 95% CIs. Depth of response (DpR) was analyzed as a continuous variable using the Kruskal–Wallis test. Its distribution was illustrated by waterfall plots, and longitudinal tumor burden changes were visualized with spaghetti plots. All tests were two-sided, with *p* < 0.05 considered statistically significant.

## 3 Results

### 3.1 Baseline Characteristics in the Overall Cohort

A total of 241 patients were included (panitumumab, *n* = 76; cetuximab, *n* = 80; bevacizumab, *n* = 85). Age, sex, and ECOG performance status were comparable across groups. Significant differences were observed in several clinicopathologic variables. Anti-EGFR groups were more likely to have left-sided tumors, whereas right-sided tumors were more common in the bevacizumab group (*p* = 0.01). MMR status differed among groups (*p* = 0.02), with a higher proportion of MSS tumors in the cetuximab group and more unknown cases in the bevacizumab group. *BRAF* mutation was more frequent in the bevacizumab group (*p* = 0.03).

Metastatic patterns were similar across groups. In contrast, primary tumor resection rates differed significantly (*p* < 0.01), being highest in the cetuximab group. Treatment initiation year also varied (*p* = 0.03), with more bevacizumab-treated patients in earlier years. Chemotherapy backbone distribution differed markedly (*p* < 0.01), with oxaliplatin-based regimens more common in the panitumumab group and irinotecan-based regimens predominating in the cetuximab and bevacizumab groups (Table 1).

**Table 1:** Baseline characteristics in the overall cohort.

Characteristic	Panitumumab (n = 76)	Cetuximab (n = 80)	Bevacizumab (n = 85)	*p* Value
**Age, years, median (IQR)**	62.0 (51.8–70.2)	60.0 (51.8–67.0)	62.0 (54.0–67.0)	0.80
**Sex, n (%)**				
Male	46 (60.5%)	56 (70.0%)	55 (64.7%)	0.46
Female	30 (39.5%)	24 (30.0%)	30 (35.3%)	
**ECOG performance status, n (%)**				
0	36 (47.4%)	40 (50.0%)	35 (41.2%)	0.72
1	31 (40.8%)	34 (42.5%)	41 (48.2%)	
2	9 (11.8%)	6 (7.5%)	9 (10.6%)	
**Tumor sidedness, n (%)**				
Left-sided	57 (75.0%)	61 (76.2%)	47 (55.3%)	
Right-sided	16 (21.1%)	19 (23.8%)	32 (37.6%)	0.01
Unknown	3 (3.9%)	0 (0.0%)	6 (7.1%)	
**MMR status, n (%)**				
MSS	55 (72.4%)	69 (86.2%)	56 (65.9%)	0.02
MSI-H	1 (1.3%)	0 (0.0%)	0 (0.0%)	
Unknown	20 (26.3%)	11 (13.8%)	29 (34.1%)	
**BRAF mutation, n (%)**				
Wild type	69 (90.8%)	74 (92.5%)	68 (80.0%)	0.03
Mutant	7 (9.2%)	6 (7.5%)	17 (20.0%)	
**Peritoneum-only metastasis, n (%)**				
No	66 (86.8%)	69 (86.2%)	64 (75.3%)	0.09
Yes	10 (13.2%)	11 (13.8%)	21 (24.7%)	
**Liver-only metastasis, n (%)**				
No	38 (50.0%)	40 (50.0%)	54 (63.5%)	0.13
Yes	38 (50.0%)	40 (50.0%)	31 (36.5%)	
**Lung-only metastasis, n (%)**				
No	72 (94.7%)	79 (98.8%)	79 (92.9%)	0.19
Yes	4 (5.3%)	1 (1.2%)	6 (7.1%)	
**Multiple-organ metastasis, n (%)**				
No	55 (72.4%)	55 (68.8%)	69 (81.2%)	0.17
Yes	21 (27.6%)	25 (31.2%)	16 (18.8%)	
**Primary tumor resection, n (%)**				
No	49 (64.5%)	27 (33.8%)	45 (52.9%)	<0.01
Yes	27 (35.5%)	53 (66.2%)	40 (47.1%)	
**Year of treatment initiation, n (%)**				
2016–2020	16 (21.1%)	18 (22.5%)	32 (37.6%)	0.03
2021–2024	60 (78.9%)	62 (77.5%)	53 (62.4%)	
**Chemotherapy backbone, n (%)**				
Oxaliplatin-based	36 (47.4%)	16 (20.0%)	16 (18.8%)	<0.01
Irinotecan-based	33 (43.4%)	53 (66.2%)	57 (67.1%)	
Others	7 (9.2%)	11 (13.8%)	12 (14.1%)	

ECOG, Eastern Cooperative Oncology Group; MMR, mismatch repair; MSS, microsatellite stable; MSI-H, microsatellite instability-high.

### 3.2 Baseline Characteristics of the Liver-Limited Subgroup

Among patients with liver-limited metastatic disease, 38 patients in the panitumumab group, 40 in the cetuximab group, and 31 in the bevacizumab group were identified (Table 2). The distribution of liver tumor burden, categorized as <10 versus ≥10 liver lesions, differed significantly among the three groups (*p* = 0.02), with the bevacizumab group having the highest proportion of patients with ≥10 liver lesions. In contrast, the largest liver metastasis diameter, metachronous presentation, and bilobar involvement did not differ significantly across groups.

**Table 2:** Baseline liver-specific characteristics of patients with liver-limited metastatic colorectal cancer according to first-line targeted therapy.

Characteristic	Panitumumab (n = 38)	Cetuximab (n = 40)	Bevacizumab (n = 31)	*p* Value
**Liver lesions**				
<10	19 (50.0%)	24 (60.0%)	8 (25.8%)	0.02
≥10	19 (50.0%)	16 (40.0%)	23 (74.2%)	
**Largest liver metastasis diameter, cm**	5.1 (3.3–8.4)	3.9 (2.4–9.7)	6.0 (3.2–7.8)	0.47
**Metachronous liver metastasis**				
No	36 (94.7%)	39 (97.5%)	31 (100.0%)	0.41
Yes	2 (5.3%)	1 (2.5%)	0 (0.0%)	
**Bilobar liver metastasis**				
No	8 (21.1%)	6 (15.0%)	3 (9.7%)	0.43
Yes	30 (78.9%)	34 (85.0%)	28 (90.3%)	

Data are presented as median (interquartile range) or n (%).

### 3.3 Survival Outcomes in the Overall Cohort

**Figure 2:**
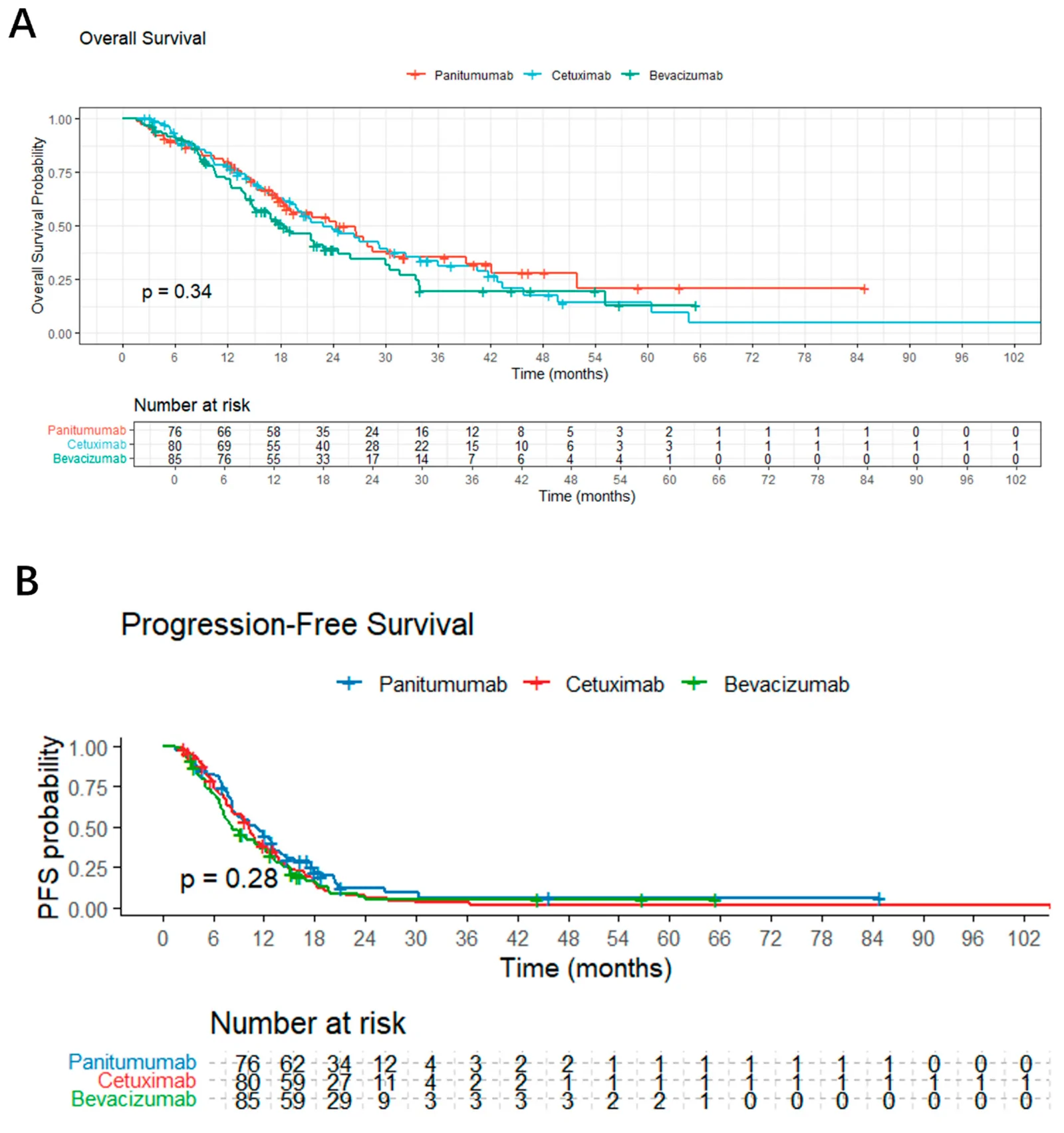
**Survival outcomes in the overall cohort.** (**A**) Kaplan–Meier curves for overall survival according to first-line treatment group. (**B**) Kaplan–Meier curves for progression-free survival according to first-line treatment group.

In the overall cohort, no significant difference in OS was observed among the panitumumab, cetuximab, and bevacizumab groups (Fig. 2A). Median OS was 24.4, 22.9, and 18.3 months, respectively (log-rank *p* = 0.34). Pairwise comparisons were also nonsignificant. Likewise, PFS did not differ significantly among the three groups (Fig. 2B). Median PFS was 11.9 months for panitumumab, 10.3 months for cetuximab, and 8.3 months for bevacizumab (log-rank *p* = 0.28). In the overall real-world cohort, differences in biologic agent selection did not translate into statistically significant survival differences.

### 3.4 Depth of Response and Treatment Activity

**Figure 3:**
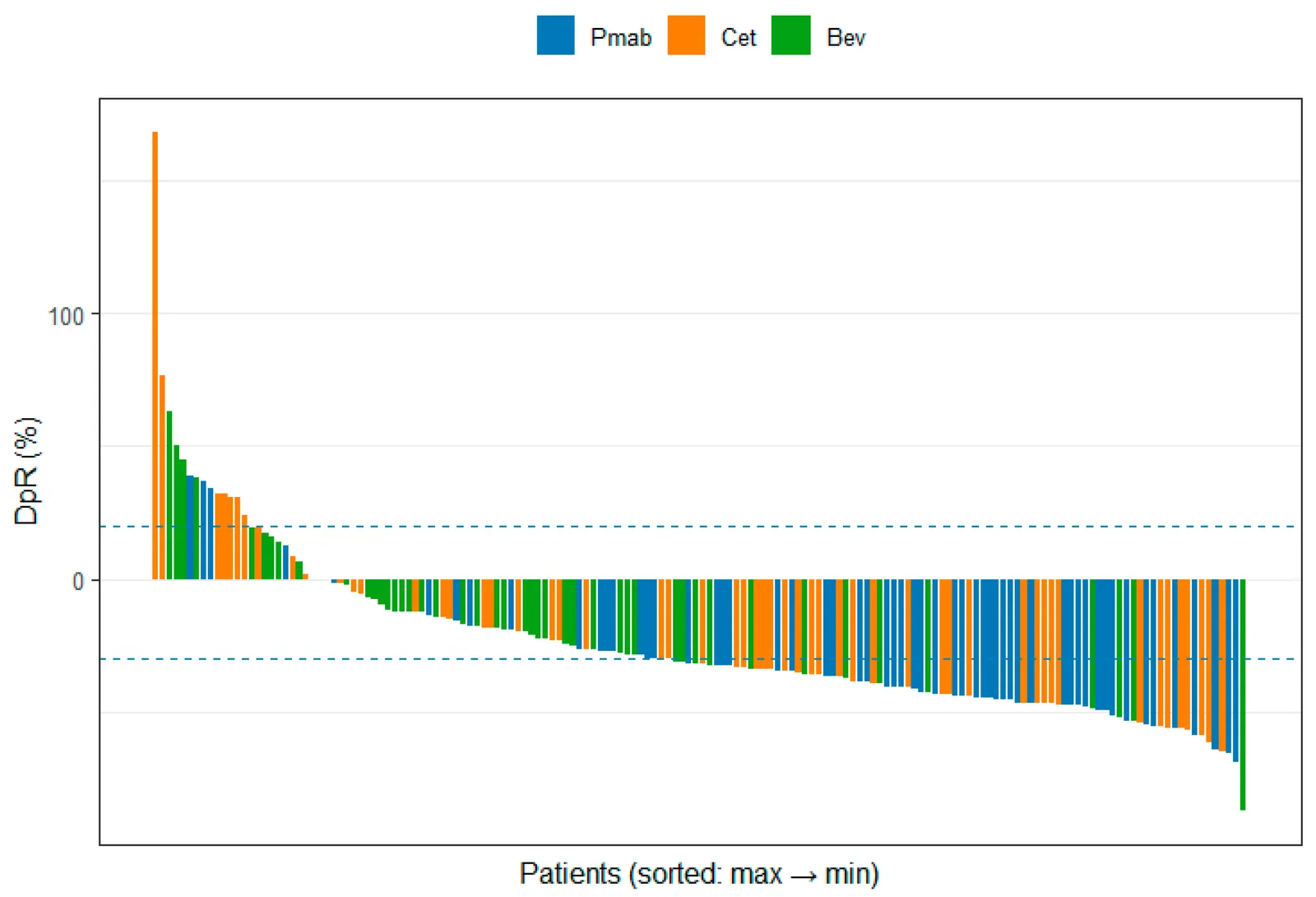
**Depth of response and longitudinal tumor change in the overall cohort.** Waterfall plot of depth of response according to treatment group.

Radiologic tumor shrinkage showed clear differences across treatments. Panitumumab achieved the deepest response, with a median DpR of −40.2%, compared with −33.4% for cetuximab and −18.7% for bevacizumab, consistent with the waterfall distribution (Fig. 3). This greater cytoreductive effect was reflected in response outcomes (Table 3). ORR was highest with panitumumab (69.7%), followed by cetuximab (50.0%) and bevacizumab (38.8%). Compared with bevacizumab, panitumumab significantly improved ORR (OR 3.63, 95% CI 1.92–6.84; *p* < 0.01), whereas cetuximab did not.

DCR was comparable across groups. In contrast, conversion surgery rates differed substantially, occurring in 34.2% of panitumumab-treated patients, 20.0% with cetuximab, and 11.8% with bevacizumab. Panitumumab was associated with a significantly higher likelihood of conversion compared with bevacizumab (OR 4.13, 95% CI 1.79–9.50; *p* < 0.01), while cetuximab showed no significant difference (Table 3). Imaging assessment characteristics were generally comparable across treatment groups. The median imaging interval ranged from 3.7 to 4.3 months, with a median of 2–3 imaging assessments performed during first-line therapy. The median time to best response (nadir) ranged from 7.0 to 8.5 months (Supplementary Table S1).

**Table 3:** Objective response rate (ORR), disease control rate (DCR), and conversion surgery rate in the whole cohort according to first-line targeted therapy.

Outcome	Comparison	Study Group n/N (%)	Bev n/N (%)	OR (95% CI)	*p* Value
ORR	Pmab vs. Bev	53/76 (69.7%)	33/85 (38.8%)	3.63 (1.92–6.84)	<0.01
ORR	Cet vs. Bev	40/80 (50.0%)	33/85 (38.8%)	1.58 (0.85–2.95)	0.16
DCR	Pmab vs. Bev	63/76 (82.9%)	67/85 (78.8%)	1.30 (0.58–2.91)	0.55
DCR	Cet vs. Bev	63/80 (78.8%)	67/85 (78.8%)	1.00 (0.46–2.20)	>0.99
Conversion rate	Pmab vs. Bev	27/76 (35.5%)	10/85 (11.8%)	4.13 (1.79–9.50)	<0.01
Conversion rate	Cet vs. Bev	16/80 (20.0%)	10/85 (11.8%)	1.88 (0.77–4.61)	0.20

Odds ratios (ORs) and 95% confidence intervals (CIs) were estimated using logistic regression, with the bevacizumab group as the reference. Pmab, panitumumab; Cet, cetuximab; Bev, bevacizumab; ORR, objective response rate; DCR, disease control rate; CI, confidence interval.

### 3.5 Survival Outcomes in Patients with Liver-Limited Metastases

**Figure 4:**
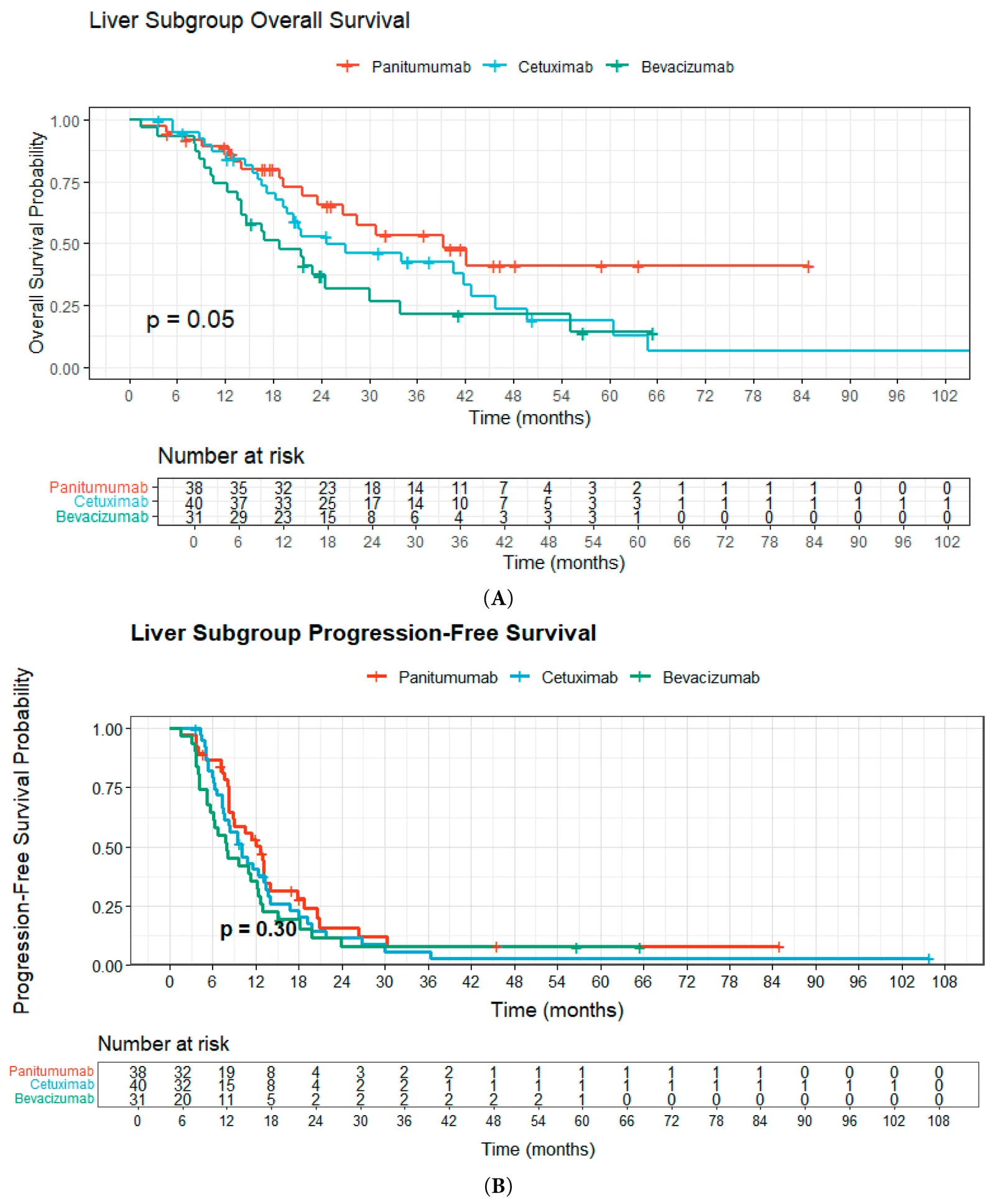
**Survival outcomes in patients with liver-limited metastases.** (**A**) Kaplan–Meier curves for overall survival according to first-line treatment group in patients with liver-limited metastatic disease. (**B**) Kaplan–Meier curves for progression-free survival according to first-line treatment group in patients with liver-limited metastatic disease.

In the liver-limited subgroup, OS showed clear separation across treatments. Median OS was 39.2 months with panitumumab, 24.7 months with cetuximab, and 18.8 months with bevacizumab, with borderline overall significance (*p* = 0.05) (Fig. 4A). In pairwise comparisons, panitumumab was associated with significantly longer OS than bevacizumab (*p* = 0.01), while other comparisons were not significant. PFS did not differ significantly among groups (median: 12.7, 10.0, and 7.9 months, respectively; *p* = 0.30) (Fig. 4B).

Consistent with the overall cohort, anti-EGFR therapy was associated with higher response rates (Table 4). ORR was 71.1% (panitumumab), 57.5% (cetuximab), and 19.4% (bevacizumab). Both panitumumab (OR 10.23, 95% CI 3.23–32.42; *p* < 0.01) and cetuximab (OR 5.64, 95% CI 1.85–17.17; *p* < 0.01) significantly improved ORR versus bevacizumab, whereas DCR was similar across groups. Conversion surgery rates were highest with panitumumab (50.0%), followed by cetuximab (35.0%) and bevacizumab (19.4%). Compared with bevacizumab, panitumumab significantly increased conversion likelihood (OR 4.17, 95% CI 1.39–12.47; *p* = 0.01), while cetuximab did not. Additional surgical outcome details are summarized in Supplementary Table S2.

**Table 4:** Objective response rate (ORR), disease control rate (DCR), and conversion surgery rate in patients with liver-limited metastatic disease according to first-line targeted therapy.

Outcome	Comparison	Study Group n/N (%)	Bev n/N (%)	OR (95% CI)	*p* Value
ORR	Pmab vs. Bev	27/38 (71.1%)	6/31 (19.4%)	10.23 (3.23–32.42)	<0.01
ORR	Cet vs. Bev	23/40 (57.5%)	6/31 (19.4%)	5.64 (1.85–17.17)	<0.01
DCR	Pmab vs. Bev	33/38 (86.8%)	27/31 (87.1%)	0.98 (0.24–4.10)	>0.99
DCR	Cet vs. Bev	32/40 (80.0%)	27/31 (87.1%)	0.59 (0.15–2.31)	0.53
Conversion rate	Pmab vs. Bev	19/38 (50.0%)	6/31 (19.4%)	4.17 (1.39–12.47)	0.01
Conversion rate	Cet vs. Bev	14/40 (35.0%)	6/31 (19.4%)	2.23 (0.73–6.79)	0.19

Odds ratios (ORs) and 95% confidence intervals (CIs) were estimated using logistic regression, with the bevacizumab group as the reference. Pmab, panitumumab; Cet, cetuximab; Bev, bevacizumab; ORR, objective response rate; DCR, disease control rate; CI, confidence interval.

### 3.6 Univariable and Multivariable Cox Regression Analysis for Overall Survival

In the overall cohort, treatment group was not independently associated with OS in multivariable analysis (Table 5). Compared with bevacizumab, neither cetuximab (HR 0.83, 95% CI 0.56–1.21; *p* = 0.33) nor panitumumab (HR 0.75, 95% CI 0.5–1.12; *p* = 0.16) showed a significant effect. Right-sided primary tumor (aHR 1.63; *p* = 0.02) and *BRAF* mutation (aHR 1.93; *p* = 0.02) were independently associated with worse OS, whereas primary tumor resection was associated with improved OS (aHR 0.68; *p* = 0.03). Other variables were not significant. First-line treatment exposure characteristics are summarized in Supplementary Table S3. Median treatment duration ranged from 9.0 to 12.0 months across groups, with a median of 10.5–12 treatment cycles administered. Maintenance therapy was more frequently observed in the panitumumab group (30.3%) than in the cetuximab (3.8%) or bevacizumab (10.6%) groups. The proportions of patients receiving second-line and third-line or later therapies are also presented in Supplementary Table S3.

In the liver-limited subgroup, treatment group remained not independently associated with OS (Table 6). Panitumumab showed a non-significant favorable trend (aHR 0.64; *p* = 0.21), while cetuximab was not associated with OS (aHR 1.38; *p* = 0.35). Within this subgroup, *BRAF* mutation (aHR 5.75; *p* < 0.01) and ≥10 liver lesions (aHR 2.57; *p* < 0.01) were independently associated with worse OS, whereas other variables were not significant.

**Table 5:** Multivariable Cox proportional hazards regression analysis for overall survival in the overall cohort. Bevacizumab was used as the reference category for treatment group comparisons.

Variable	Univariable HR	95% CI	*p*	Multivariable HR	95% CI	*p*
Cet vs. Bev	0.83	0.56–1.21	0.33			
Pmab vs. Bev	0.75	0.50–1.12	0.16			
Age ≥65	1.41	1.01–1.96	0.04	1.36	0.93–1.98	0.11
Female	1.01	0.72–1.41	0.97			
ECOG 1 vs. 0	1.2	0.85–1.70	0.31			
ECOG 2 vs. 0	1.64	0.96–2.79	0.07	1.42	0.77–2.62	0.26
Right-sided vs. Left-sided	1.9	1.34–2.72	<0.001	1.63	1.08–2.47	0.02
BRAF mutation	2.21	1.34–3.64	0.002	1.93	1.11–3.35	0.02
Peritoneal metastasis	2.4	1.60–3.60	<0.001	1.85	0.98–3.49	0.06
Liver-only metastasis	0.58	0.42–0.81	0.001	0.76	0.43–1.36	0.36
Multiple-organ metastasis	1.17	0.80–1.70	0.42			
Primary resection	0.81	0.58–1.12	0.19	0.68	0.48–0.97	0.03
Treatment start ≥2021	0.84	0.59–1.18	0.3			
Oxaliplatin-based	0.97	0.69–1.37	0.87			

HR, hazard ratio; CI, confidence interval; Pmab, panitumumab; Cet, cetuximab; Bev, bevacizumab; ECOG, Eastern Cooperative Oncology Group.

**Table 6:** Cox regression analysis for overall survival in the liver-limited subgroup.

Variable	Adjusted HR	95% CI	*p* Value
Cet vs. Bev	1.38	0.71–2.69	0.35
Pmab vs. Bev	0.64	0.31–1.29	0.21
Age ≥65	1.48	0.80–2.75	0.22
Female	0.99	0.57–1.71	0.98
ECOG ≥1	1.23	0.70–2.15	0.48
Right-side tumor	0.98	0.45–2.11	0.95
BRAF mutation	5.75	1.77–18.68	<0.01
Primary tumor resection	0.89	0.48–1.65	0.72
Oxaliplatin-based	0.65	0.32–1.32	0.24
Liver lesions ≥10	2.57	1.37–4.84	<0.01
Diameter ≥5 cm	1.34	0.74–2.44	0.33
Bilobar involvement	1.61	0.63–4.14	0.32
Metachronous liver metastasis	0.58	0.13–2.73	0.49

HR, hazard ratio; CI, confidence interval; Pmab, panitumumab; Cet, cetuximab; Bev, bevacizumab; ECOG, Eastern Cooperative Oncology Group.

### 3.7 Pooled Anti-EGFR Versus Bevacizumab Analysis

To address the potential impact of limited sample size in pairwise comparisons, an exploratory pooled analysis combining panitumumab and cetuximab into a single anti-EGFR group was performed. Compared with bevacizumab, anti-EGFR therapy was associated with higher ORR (59.6% vs. 38.8%, *p* < 0.01) and conversion rates (27.6% vs. 11.8%, *p* < 0.01), while DCR, PFS, and OS were not significantly different (Supplementary Table S4).

## 4 Discussion

Our findings need to be considered in the context of current first-line treatment strategies for *RAS* wild-type metastatic colorectal cancer, in which anti-EGFR therapy remains an important option, particularly for left-sided tumors. Prior randomized trials and pooled analyses have shown that EGFR-directed treatment can achieve higher response rates and greater tumor shrinkage than bevacizumab-based therapy in molecularly selected populations, although the survival benefit is influenced by tumor sidedness and other baseline factors [4,5,6,20]. In this setting, treatment selection is not based on survival alone. The likelihood of achieving rapid and substantial tumor regression is also relevant, especially when metastasis-directed treatment may still be feasible.

Direct first-line comparative data between cetuximab and panitumumab remain limited. Although the ASPECCT trial showed similar efficacy in the later-line setting [12], this does not fully address their relative role as induction therapy. In our cohort, a trend favoring panitumumab was most apparent in patients with liver-limited disease; however, this did not translate into a statistically significant difference compared with the cetuximab group. Although direct evidence specifically supporting panitumumab as the preferred biologic in this setting remains limited, prior studies have demonstrated robust cytoreductive activity with anti-EGFR–based therapy and highlighted its role in conversion-oriented strategies for colorectal liver metastases [17,21,22,23,24,25]. 

Although selection bias related to patient eligibility and resectability cannot be completely excluded, the observed trend toward greater cytoreduction in liver-limited disease remains biologically plausible. In liver-limited disease, treatment response has implications beyond disease control, as the magnitude of tumor shrinkage may directly influence resectability and the feasibility of metastasis-directed local therapy. In addition, colorectal liver metastases allow for more standardized and reproducible radiologic assessment, particularly with contrast-enhanced CT and MRI, facilitating objective evaluation of tumor response over time. Notably, organ-specific analyses have suggested that liver metastases may exhibit greater sensitivity to targeted therapy, with deeper tumor shrinkage more strongly associated with survival compared with extrahepatic sites. Consistent with these observations, previous studies have demonstrated that early tumor shrinkage and depth of response are closely associated with long-term outcomes in patients receiving anti-EGFR–based therapy [26,27,28,29]. Nevertheless, the absence of a significant difference between panitumumab and cetuximab in our analysis suggests that the observed benefit should be interpreted as a treatment signal rather than definitive evidence of superiority. 

Although cetuximab and panitumumab are both anti-EGFR monoclonal antibodies, they are not biologically identical [30]. Cetuximab is a chimeric IgG1 antibody with antibody-dependent cellular cytotoxicity activity, whereas panitumumab is a fully human IgG2 antibody with minimal immune effector function. These differences may translate into distinct pharmacologic and safety profiles, as suggested by real-world pharmacovigilance data [31,32,33,34,35]. Beyond these pharmacologic differences, cetuximab may also exert immune-mediated antitumor activity through antibody-dependent cellular cytotoxicity (ADCC), a mechanism that is largely absent with panitumumab [36,37]. Previous translational studies have suggested that host-related factors, including Fcγ receptor polymorphisms, may influence cetuximab efficacy and clinical outcome [38]. Although such biomarkers were not available in the present cohort, these findings highlight the biological complexity underlying anti-EGFR treatment response and suggest that panitumumab and cetuximab may not be fully interchangeable in all clinical settings. In this context, the greater tumor shrinkage observed with panitumumab in our cohort may reflect differences in patient selection, tumor biology, or potential agent-specific effects. However, given the retrospective design and limited sample size, these findings should not be interpreted as definitive evidence that panitumumab and cetuximab are clinically non-interchangeable. Analyses involving conversion surgery are inherently vulnerable to immortal time bias, as patients must survive and remain progression-free long enough to undergo local treatment. Therefore, the observed association between conversion surgery and improved survival outcomes should be interpreted cautiously and regarded as exploratory rather than causal. In particular, patients selected for anti-EGFR–based therapy may have been considered more suitable candidates for conversion-oriented treatment strategies at baseline, which could have influenced both response-related and surgical outcomes.

Our multivariable analyses indicate that primary tumor biology, rather than biologic choice alone, remained a major determinant of survival. In the overall cohort, right-sided primary tumor location and *BRAF* mutation emerged as the most adverse independent factors for OS, whereas in the liver-limited subgroup, *BRAF* mutation and a high hepatic tumor burden, reflected by the presence of ≥10 liver metastases, were independently associated with inferior survival. These findings are consistent with prior evidence showing that right-sided *RAS* wild-type tumors have a distinctly worse prognosis and derive limited benefit from anti-EGFR-based treatment compared with left-sided disease, while adverse molecular features such as *BRAF* mutation and greater liver tumor burden continue to strongly influence outcome even in patients considered for active systemic and liver-directed treatment. Accordingly, patients with right-sided primary tumors or *BRAF*-mutated disease should not be viewed as candidates for a uniform EGFR plus chemotherapy approach. These findings further support the importance of considering tumor sidedness, molecular profile, metastatic pattern, and resectability when selecting systemic treatment strategies for patients with metastatic colorectal cancer. Instead, these patients are more appropriately managed through a multidisciplinary, personalized treatment strategy, integrating tumor biology, metastatic pattern, resectability, and the availability of biomarker-directed therapy. In particular, the BREAKWATER trial has now established encorafenib plus cetuximab and mFOLFOX6 as a more appropriate first-line option for *BRAF V600E*-mutated metastatic colorectal cancer, highlighting that this subgroup should no longer be approached with standard EGFR plus chemotherapy alone. In addition, the emergence of resistance to anti-EGFR therapy remains a major clinical challenge, with multiple molecular mechanisms contributing to treatment failure in *RAS* wild-type disease [39]. 

This study has several limitations. It was a single-center retrospective analysis, and treatment allocation was determined in routine practice rather than by randomization. The sample size was modest, particularly in the liver-limited subgroup, which limited the precision of pairwise and weighted comparisons. Baseline characteristics were also unevenly distributed across treatment groups, including tumor sidedness, *BRAF* mutation frequency, primary tumor resection, treatment era, chemotherapy backbone, and liver tumor burden. In addition, imaging intervals and multidisciplinary decisions regarding conversion surgery were not fully standardized. Furthermore, conversion surgery is not a completely objective endpoint and may be influenced by institutional practice patterns, multidisciplinary decision-making, surgeon preference, and the timing of radiologic reassessment. Accordingly, these findings should be regarded as hypothesis-generating and warrant confirmation in larger multicenter cohorts and prospective conversion-oriented studies. Although imaging reassessment was generally performed according to institutional and guideline-based follow-up schedules, variation in imaging frequency and follow-up duration is unavoidable in retrospective real-world studies. Additional restriction of the cohort using fixed imaging landmarks or minimum follow-up requirements would have substantially reduced the available sample size and statistical power. Therefore, potential ascertainment bias related to imaging timing cannot be completely excluded and should be considered when interpreting response-related endpoints, including DpR and time-to-nadir analyses. In addition, response assessment in the present study was based on conventional RECIST 1.1 criteria. Emerging imaging approaches, including radiomics and machine-learning–based analyses, may provide additional information regarding tumor heterogeneity, growth patterns, recurrence risk, and treatment sensitivity. Future studies incorporating advanced imaging biomarkers may further refine response prediction and patient selection in conversion-oriented treatment strategies. Importantly, although DpR has been associated with conversion potential and favorable long-term outcomes in previous studies [40], it should not be interpreted as a direct surrogate for survival benefit. Tumor shrinkage reflects both treatment effect and underlying tumor biology, and the absence of a significant OS advantage in the present study suggests that DpR alone may be insufficient to fully capture long-term clinical outcomes. 

Detailed chemotherapy dose intensity metrics were not consistently available in this retrospective cohort. Therefore, the potential impact of dose reductions, treatment delays, and relative dose intensity on clinical outcomes could not be fully evaluated. MMR status was unavailable in a proportion of patients because MMR testing was not routinely performed throughout the entire study period and was generally obtained based on physician-directed clinical indications. Consequently, the potential influence of MMR status on treatment selection and clinical outcomes could not be fully assessed. Although chemotherapy backbone was incorporated into the multivariable models as a prespecified covariate, residual confounding related to treatment selection and regimen choice cannot be completely excluded. Another limitation is the lack of longitudinal molecular monitoring. The present study relied primarily on baseline molecular characteristics, whereas resistance to anti-EGFR therapy is increasingly recognized as a dynamic process driven by clonal evolution, including the emergence of secondary RAS mutations and EGFR extracellular domain alterations. Because serial circulating tumor DNA or repeat molecular profiling was not routinely available, the impact of acquired resistance mechanisms on treatment response and survival could not be evaluated. 

An additional source of potential bias arises from reimbursement policies. In Taiwan, panitumumab was not reimbursed until 2018, whereas cetuximab and bevacizumab were available earlier. As a result, patients treated between 2016 and 2018 were more likely to receive cetuximab or bevacizumab, while panitumumab use was largely confined to later years. This temporal difference may have introduced confounding related to treatment era, patient selection, and evolving clinical practice patterns. Furthermore, treatment allocation in this real-world setting was influenced not only by reimbursement constraints but also by physician preference and multidisciplinary decision-making. In addition, evolving multidisciplinary strategies, improvements in surgical techniques, and increasing experience with conversion-oriented treatment approaches over time may also have influenced clinical outcomes independently of the biologic agent administered. Consequently, the baseline characteristics of the three treatment groups were not fully comparable, and residual confounding cannot be excluded despite multivariable adjustment.

## 5 Conclusion

In summary, panitumumab-based first-line therapy was associated with greater tumor shrinkage and higher conversion rates in this real-world cohort of patients with *RAS* wild-type metastatic colorectal cancer, particularly among those with liver-limited disease. These findings suggest that greater cytoreduction may be associated with increased opportunities for local treatment in selected patients. However, given the retrospective design, baseline imbalances, treatment-selection bias, and the influence of multidisciplinary decision-making on conversion eligibility, these observations should be interpreted cautiously and considered hypothesis-generating rather than definitive evidence of comparative treatment superiority. Further prospective studies are warranted to validate these findings and clarify their impact on long-term survival outcomes.

## Data Availability

The data used to support the findings of the present study are available from the corresponding authors upon request.

## Supplementary Materials

The supplementary material is available online at https://www.techscience.com/doi/10.32604/or.2026.085229/s1.
