# Supplementary material for: Differential Tumor Response and Conversion Outcomes Associated with First-Line Biologic Strategies in Liver-Limited RAS Wild-Type Metastatic Colorectal Cancer
Source: Oncol Res. 2026 Aug 13;34(9):23. doi: 10.32604/or.2026.085229 (PMC13494483; doi:10.32604/or.2026.085229)
Supplement: Supplementary file 1 [file OncolRes-34-85229-s001.zip › TSP_OR_85229-s001.docx]

**Supplementary Table S1:** Response outcomes among patients with evaluable imaging data.

| **Variable** | **Pmab (n = 59)** | **Cet (n = 54)** | **Bev (n = 47)** |
| --- | --- | --- | --- |
| Median imaging interval, months | 3.7 | 4.3 | 3.8 |
| Median number of imaging assessments during first-line therapy | 3 | 2 | 2 |
| Median time to best response (nadir), months | 7 | 8.5 | 8.4 |

Pmab, panitumumab; Cet, cetuximab; Bev, bevacizumab.

Patients without available post-treatment imaging or measurable target lesions were excluded from this analysis. Not evaluable imaging data, 81 (33.6%)

**Supplementary Table S2:** Surgical and perioperative outcomes among patients undergoing conversion surgery.

| **Variable** | **Pmab** | **Cet** | **Bev** |
| --- | --- | --- | --- |
| Conversion surgery, n | 19 | 14 | 6 |
| Time to surgery, median months (range) | 6.2 (3.2–14.1) | 6.2 (2.7–13.4) | 7.2 (2.5–12.8) |
| Minor hepatectomy, n(%) | 12 (63.1%) | 6 (42.6%) | 2 (33.3%) |
| Major hepatectomy, n(%) | 6 (31.6%) | 8 (57.4%) | 3 (50.0%) |
| Radiofrequency ablation (RFA), n(%) | 1 (5.3%) | 0 (0%) | 1 (16.7%) |
| R0 resection, n(%) | 9 (47.4%) | 4 (28.6%) | 3 (50.0%) |
| R1 resection, n(%) | 10 (52.6%) | 10 (71.4%) | 3 (50.0%) |
| Major postoperative complication, n(%) | 3 (15.8%) | 3 (21.4%) | 2 (33.3%) |
| Extrahepatic disease at surgery, n(%) | 0 (0%) | 0 (0%) | 0 (0%) |
| **Recurrence pattern, n(%)** |  |  |  |
| Liver recurrence | 14 (73.7%) | 6 (42.6%) | 2 (33.3%) |
| Extrahepatic recurrence | 1 (5.3%) | 5 (35.7%) | 0 (0%) |

Pmab, panitumumab; Cet, cetuximab; Bev, bevacizumab; RFA, radiofrequency ablation.

**Supplementary Table S3:** First-line treatment exposure characteristics and subsequent therapy distribution.

| **Variable** | **Pmab (n = 76)** | **Cet (n = 80)** | **Bev (n = 85)** |
| --- | --- | --- | --- |
| Median number of treatment cycles | 10.5 | 11 | 12 |
| Median duration of first-line therapy, months | 12 | 10.6 | 9 |
| Maintenance therapy, n (%) | 23 (30.3%) | 3 (3.8%) | 9 (10.6%) |
| Subsequent therapy, n (%) |  |  |  |
| Received second-line therapy | 31 (40.8%) | 24 (30.0%) | 24 (28.2%) |
| Received third-line or later therapy | 15 (19.7%) | 25 (31.3%) | 27 (31.8%) |

Pmab, panitumumab; Cet, cetuximab; Bev, bevacizumab.

**Supplementary Table S4:** Pooled anti-EGFR versus bevacizumab analysis.

| **Outcome** | **Anti-EGFR**  **(n = 156)** | **Bevacizumab**  **(n = 85)** | **P value** |
| --- | --- | --- | --- |
| ORR, n (%) | 93 (59.6%) | 33 (38.8%) | 0.002 |
| DCR, n (%) | 126 (80.8%) | 67 (78.8%) | 0.71 |
| Conversion surgery, n (%) | 43 (27.6%) | 10 (11.8%) | 0.005 |
| Median PFS, months | 10.3 | 8.3 | 0.27 |
| Median OS, months | 23.9 | 18.3 | 0.17 |

ORR, objective response rate; DCR, disease control rate; PFS, progression-free survival; OS, overall survival; anti-EGFR, epidermal growth factor receptor inhibitor.
